# High-energy blunt pelvic ring injury: dataset of patients and injury characteristics from a severely injured patients’ registry.

**DOI:** 10.1016/j.dib.2022.108740

**Published:** 2022-11-09

**Authors:** Silvia Valisena, Anna-Eliane Abboud, Elisabeth Andereggen, Alexandre Ansorge, Axel Gamulin

**Affiliations:** a Division of Orthopaedic and Trauma Surgery, University Hospitals of Geneva, 4 Rue Gabrielle-Perret-Gentil, 1205, Geneva, Switzerland; b Division of Emergency Medicine, University Hospitals of Geneva, 4 Rue Gabrielle-Perret-Gentil, CH-1205 Geneva, Switzerland

**Keywords:** Pelvic region, Pelvic injuries, Blunt injury, Trauma severity indices

## Abstract

Since mid-2013, data on high-energy trauma patients admitted to the Emergency Department of the University Hospitals of Geneva, Switzerland, are prospectively recorded in a dedicated registry. This includes data on patients with high-energy blunt pelvic ring injuries (PRI), defined as closed fracture of the pelvic ring following falls from a height >1 m, road traffic accidents, sport, crush, farm and industrial injuries. The registry was screened for patients aged ≥16 years with high-energy blunt PRI admitted to the aforementioned academic level I trauma center between 2014.01.01 et 2019.12.31, to assess the outcome of the institutional PRI management protocol. Data on 195 patients were collected and analyzed for this purpose [1]. The dataset “patients’ demographic and injury characteristics” provides the raw demographics and Abbreviated Injury Scale (AIS) of these 195 patients. These data can contribute to the knowledge of patients’ demographics and injury characteristics of high-energy blunt PRI patients.

**Specifications Table**

**Table:** 

Subject	Orthopaedics, sports medicine and rehabilitation; Surgery
Specific subject area	Demographic and injury characteristics of patients with high-energy blunt pelvic ring injuries.
Type of data	Tables
How the data were acquired	The dataset was obtained by screening the Severely Injured Patients’ Registry (SIPR) of the University Hospitals of Geneva for patients with high-energy blunt pelvic ring injuries (PRI), according to the eligibility criteria mentioned under the “description of data collection” section. The dataset reports the age, sex, pelvic fracture classification, Abbreviated Injury Scale (AIS), and Injury Severity Score (ISS) of a cohort of 195 patients screened from the SIPR.
Data format	Raw
Description of data collection	The eligibility criteria for the dataset were: -Inclusion: 1) high-energy blunt PRI; 2) admission between 2014.01.01 and 2019.12.31; 3) age ≥16 years at admission. -Exclusion: 1) death before admission; 2) prior treatment in another institution; 3) institutional protocol not followed. The dataset collects demographics, fracture classification, ISS and AIS scores of eligible SIPR patients.
Data source location	Institution: University Hospitals of Geneva (HUG). City/Town/Region: Geneva, Canton de Genève, Switzerland.
Data accessibility	Repository name: Mendeley.com Data identification number: DOI:10.17632/55fjm3zctn.2 Direct URL to data: https://data.mendeley.com/datasets/55fjm3zctn The reuse of the repository data requires approval from both the senior author Dr. PD Axel Gamulin, and the Commission Cantonale d'Ethique de la Recherche, République et Canton de Genève (Ethics Committee). Access to data of the SIPR not included in the repository may be discussed with the Senior Author upon reasonable request. Each new study project necessitates a new Ethics Committee approval.
Related research article	S. Valisena, A-E Abboud, E. Andereggen, A. Ansorge, A. Gamulin, Management of high-energy blunt pelvic ring injuries: a retrospective cohort study evaluating an institutional protocol, Injury. 13 (2022) 1-8. doi:10.1016/j.injury.2022.09.020.

## Value of the Data

• The management of high-energy PRI patients is not standardized worldwide. Knowledge of patients’ demographics and injury characteristics in high-energy blunt PRI is key to the development and standardization of high-energy PRI patients’ management protocols. This dataset shows patients’ demographics and injury characteristics of the study by Valisena et al. assessing the outcome of an institutional high-energy blunt PRI management protocol [1].
• Researchers and clinicians in the orthopedic, emergency and epidemiologic fields and with an interest in high-energy blunt PRI can benefit from these data.
• Reuse of the repository dataset linked to the present article requires the approval from both the senior author Dr. PD Axel Gamulin, and the Commission Cantonale d'Ethique de la Recherche, République et Canton de Genève (Ethics Committee).

## 1 Objective

Multiple predictors of mortality for high-energy PRI patients are studied in the literature, such as: massive bleeding, age, gender, fracture type, concomitant injuries, ISS [2]. The aim of the dataset is to show age, gender, fracture type, ISS and AIS in the studied patient population.

## 2 Data Description

Data shown in Tables 1 and 2 describe the injury characteristics and demographic of a cohort of 195 patients screened from the SIPR, studied by Valisena et al [1].

**Table 1:** Patients’ demographic and injury characteristics

Case Number	Age (years)	Sex (0=Male, 1=Female)	AO OTA Classification	ISS	Highest AIS head and neck	Highest AIS face	Highest AIS chest	Highest AIS abdomen	Highest AIS pelvis extremity	Highest AIS external
1	22	1	61B1.1b,e	20	4	0	0	0	2	0
2	18	1	61A3	14	1	0	3	0	2	0
3	55	1	61A3	13	3	0	0	0	2	0
4	38	1	61A2.2	9	0	0	0	0	3	0
5	41	0	61B3.1d,g	29	0	0	3	2	4	0
6	58	0	61C1.3b	50	1	0	3	4	5	0
7	26	1	61B2.1a,e	9	0	0	0	0	3	0
8	48	1	61B3.1a	66	5	2	4	3	5	0
9	68	1	61B1.1	34	3	0	4	3	2	0
10	44	0	61A3	38	5	0	3	2	2	0
11	71	0	61B3.3a	38	1	0	3	2	5	0
12	76	1	61B2.1a,e	27	3	0	3	0	3	0
13	20	0	61B3.1d	50	4	0	5	3	3	0
14	43	0	61B1.1b,e	22	0	2	3	0	3	0
15	54	0	61B2.2b,e	27	3	3	3	0	3	0
16	34	0	61C1.3b	25	0	0	3	0	4	0
17	45	0	61B2.3d	13	0	0	0	2	3	0
18	33	0	61A3	17	3	0	2	2	2	0
19	35	0	61B1.1a	22	2	0	3	2	3	0
20	75	0	61A1.1	17	0	0	3	2	2	0
21	48	1	61B2.1a,e	17	2	0	2	0	3	0
22	56	1	61B2.1a	18	3	0	0	0	3	0
23	25	0	61C1.2a,j	13	0	0	0	2	3	0
24	39	1	61A3	29	3	0	4	2	2	2
25	57	0	61C2.3d,m	20	0	0	0	2	4	0
26	46	0	61B1.1	22	2	0	3	2	3	0
27	72	1	61B3.2a	27	3	2	3	2	3	0
28	26	0	61C1.3d	29	0	0	3	2	4	0
29	87	1	61B2.2a,e	17	0	0	2	2	3	0
30	69	1	61A3	45	5	0	4	0	2	0
31	34	0	61B2.1b,e	21	2	1	0	0	4	0
32	19	1	61B2.1b,d,e	25	0	0	4	0	3	0
33	50	0	61B2.1b,e	13	0	0	0	2	3	0
34	19	1	61B2.1b	16	0	0	0	0	4	0
35	69	1	61A1.1	17	2	2	2	0	3	0
36	45	1	61A2.1	29	2	0	3	4	2	0
37	35	0	61A2.1	22	3	3	0	1	2	0
38	74	1	61C1.3b,e	43	3	3	0	2	5	0
39	87	0	61B3.2a	22	2	0	3	0	3	0
40	61	0	61A2.2	14	1	0	3	0	2	0
41	41	0	61C3.3d,h,j	50	4	0	3	3	5	0
42	39	1	61C3.3b,d,e,f,j	43	3	2	2	3	5	0
43	39	1	61B2.1a,e	13	2	0	0	0	3	0
44	21	1	61B2.1b,e	34	0	3	2	3	4	0
45	42	1	61B2.1b,e	10	0	0	0	0	3	1
46	39	0	61B1.1	34	4	3	3	3	3	0
47	25	0	61A2.2	29	2	0	4	3	2	0
48	55	0	61B3.1a,d,e,f	29	0	0	0	2	5	0
49	27	1	61B3.2a,e	17	0	0	2	2	3	0
50	76	0	61A2.1	17	2	0	3	0	2	0
51	63	0	61B2.1a	17	2	0	2	2	3	0
52	55	0	61C2.3d,m	29	0	0	3	2	4	0
53	37	0	61B3.3d	24	2	0	1	2	4	0
54	49	0	61C1.3a	20	0	0	0	2	4	0
55	22	0	61B2.2a	17	0	0	2	2	3	0
56	18	0	61A1.1	41	3	1	4	4	2	0
57	21	1	61B2.1a	34	0	0	3	0	5	0
58	48	0	61B2.1b,e	34	5	0	0	0	3	0
59	24	0	61C3.3a,e	34	3	0	3	2	4	0
60	23	1	61A3	45	0	0	4	5	2	0
61	25	1	61B3.2b,e	43	5	0	3	3	3	0
62	27	1	61B2.2c	22	3	0	2	0	3	0
63	32	0	61B3.1a	50	5	1	3	4	3	0
64	52	1	61B2.2b,e	18	3	0	0	0	3	0
65	21	0	61B2.1	38	2	0	5	0	3	0
66	63	0	61B3.2	27	3	3	0	2	3	0
67	22	1	61B3.2	27	3	0	2	3	3	0
68	36	0	61B2.1a,e	22	0	0	3	2	3	0
69	24	1	61B2.2a,e	54	5	0	2	0	5	0
70	46	0	61B2.3d	13	0	0	0	2	3	0
71	71	0	61C1.2d,j	24	0	0	2	2	4	0
72	28	0	61B3.2b,e	43	5	0	3	3	3	0
73	26	1	61C3.3a,e	29	0	0	3	2	4	0
74	55	0	61A3	22	0	0	3	3	2	0
75	17	0	61B2.1a,d,e,f	29	2	0	3	0	4	0
76	29	1	61C1.3b,e	21	0	1	0	2	4	0
77	44	0	61B2.2a	9	0	0	0	0	3	0
78	59	0	61B2.2a	33	1	0	4	0	4	0
79	81	1	61B1.1c,e	43	5	0	3	0	3	0
80	37	1	61B2.1a,e	17	2	0	2	2	3	0
81	49	1	61B1.2a	13	2	0	0	0	3	0
82	68	0	61A3	38	5	0	3	0	2	0
83	56	1	61B2.1a,e	43	5	3	3	0	3	0
84	52	1	61B2.2c,e	36	0	0	4	2	4	0
85	27	0	61A2.2	22	0	0	3	3	2	0
86	69	0	61B2.2a,e	29	0	0	3	2	4	0
87	28	0	61B2.2a,e	43	1	0	3	5	3	0
88	30	1	61B1.1a,e	5	0	0	1	0	2	0
89	33	0	61B1.1a,e	59	3	0	5	5	3	0
90	58	0	61B1.1a	17	2	0	3	0	2	0
91	51	0	61B1.1a	5	0	0	0	1	2	0
92	18	0	61B2.2a	27	0	0	3	3	3	0
93	54	0	61A1.3	34	0	0	4	3	3	0
94	78	0	61B3.2b,e	34	3	0	3	0	4	0
95	48	1	61B2.3c	27	3	0	2	3	3	0
96	76	0	61B2.2a,e	41	4	4	3	0	3	0
97	56	1	61B3.2a,e	18	0	0	3	0	3	0
98	52	1	61C3.3	34	3	0	2	3	4	0
99	53	1	61C1.3a,e	24	0	0	2	2	4	0
100	20	0	61B2.1a	20	0	0	0	2	4	0
101	49	0	61C3.3	24	2	2	0	2	4	0
102	49	0	61B2.3d	10	1	0	0	0	3	0
103	53	1	61B2.3a,d	38	0	0	3	2	5	0
104	26	0	61A3	17	3	2	0	2	2	0
105	20	1	61B3.1a	13	0	0	0	2	3	0
106	80	0	61B2.2a	18	0	0	3	0	3	0
107	50	1	61B2.1a,e	14	0	0	0	2	3	1
108	22	0	61B2.1b,e	22	0	2	3	0	3	0
109	32	1	61B2.2a,d	41	0	0	3	4	4	0
110	57	0	61B3.1b,e	16	0	0	0	0	4	0
111	38	0	61B2.1a	19	1	0	3	0	3	0
112	31	1	61A2.3	17	0	0	3	2	2	1
113	30	0	61C2.3b,e,m	43	3	1	3	2	5	0
114	61	0	61A2.1	22	2	3	2	3	2	0
115	66	0	61B2.2a	22	0	2	3	0	3	0
116	26	0	61C1.1d	17	1	0	0	0	4	0
117	79	0	61A2.1	22	3	0	3	0	2	1
118	51	0	61B3.3b,d,e	9	0	0	0	0	3	0
119	82	0	61B2.3d	25	0	0	3	0	4	0
120	28	0	61A3	17	0	0	3	2	2	0
121	42	1	61B2.1c	9	0	0	0	0	3	0
122	41	0	61A2.1	34	4	1	3	3	2	0
123	41	1	61C3.3b,e	57	2	0	4	4	5	0
124	44	1	61B2.2b,e	27	3	0	3	2	3	0
125	67	1	61B1.1a,e	9	0	0	0	0	3	0
126	48	1	61B2.1b,e	9	0	0	0	0	3	0
127	41	0	61B2.2b,e	16	0	0	0	0	4	0
128	16	1	61A2.2	4	0	0	0	0	2	0
129	16	1	61B2.1a,e	17	2	0	2	0	3	0
130	90	1	61B2.2c,e	19	1	0	3	0	3	0
131	52	0	61C1.2a,d,e	30	0	0	1	2	5	0
132	37	0	61B2.1a	17	0	0	2	2	3	0
133	84	1	61A1.1	17	0	0	3	2	2	0
134	37	0	61C1.3a,e	29	0	0	3	2	4	1
135	38	0	61A3	8	0	0	0	2	2	0
136	28	0	61C2.1b,e,j,k	24	0	0	2	2	4	1
137	25	0	61A1.1	6	1	0	1	0	2	0
138	20	0	61A2.1	17	3	0	2	0	2	1
139	60	0	61B2.3a,d	34	0	0	3	3	4	0
140	63	0	61B2.3d	24	0	0	2	2	4	0
141	71	1	61B2.1a	50	5	4	3	2	3	1
142	24	1	61B1.1a	14	2	0	0	0	3	1
143	46	0	61B2.1a	66	5	3	5	4	3	0
144	26	1	61C2.3c,m	50	3	0	4	0	5	1
145	43	0	61C2.1a,k	16	0	0	0	0	4	0
146	50	0	61B2.1a	38	0	0	5	2	3	1
147	30	0	61A2.1	27	3	0	3	3	2	1
148	68	1	61B2.1b	41	4	1	2	3	4	0
149	23	1	61A2.1	50	5	2	4	3	2	0
150	75	1	61B2.1a	22	3	0	0	2	3	0
151	34	1	61B1.1b	19	0	0	0	3	3	1
152	26	1	61C1.3a	66	5	2	4	4	5	0
153	53	0	61A3	4	0	0	0	0	2	0
154	42	0	61B2.1c,e	34	4	3	3	0	3	1
155	43	1	61B2.2b,e	29	2	0	3	0	4	0
156	21	0	61B1.1c	29	0	0	2	4	3	1
157	44	1	61B2.1b	34	0	0	3	4	3	0
158	22	1	61B3.1b,e	32	0	0	4	0	4	0
159	29	0	61B3.3b,d	20	0	0	0	2	4	0
160	49	1	61B2.1b,e	16	0	0	0	0	4	0
161	77	1	61B2.1b,e	22	2	0	3	0	3	1
162	35	0	61B1.1a	13	0	0	0	2	3	0
163	27	0	61B2.1b	20	0	0	0	2	4	0
164	66	1	61B1.1a,e	17	2	2	0	0	3	0
165	56	1	61B2.2a,e	10	1	0	0	0	3	0
166	72	1	61A3	17	3	0	2	0	2	0
167	52	1	61B2.1b,e	10	0	0	0	0	3	1
168	52	0	61C2.3c,d,e,m	41	2	0	3	4	4	1
169	91	0	61B2.1a	27	3	0	3	0	3	2
170	62	1	61B3.1a,e	24	2	0	2	0	4	0
171	29	0	61B2.1a	29	2	1	3	2	4	2
172	40	1	61B2.1b,e	9	0	0	0	0	3	0
173	53	0	61C2.2c,d,m	38	0	0	3	2	5	0
174	24	0	61B3.1a,e	9	0	0	0	0	3	0
175	35	1	61A3	24	0	0	2	4	2	0
176	49	0	61B3.3d	27	3	0	3	2	3	0
177	29	0	61B2.3b,d	24	0	0	2	2	4	0
178	83	1	61A1.1	21	1	0	4	0	2	1
179	64	1	61B2.1a	13	0	0	0	2	3	0
180	60	0	61C3.2c,d,e,h	66	5	0	4	2	5	0
181	34	1	61B2.1a,e	22	3	2	0	2	3	1
182	61	1	61B2.1a	10	0	0	0	0	3	1
183	83	1	61B2.1c,e	66	5	0	4	0	5	0
184	68	0	61B2.1b,e	18	0	0	3	0	3	0
185	44	1	61B2.1a,e	17	2	0	0	2	3	1
186	16	0	61A2.1	50	5	0	4	2	3	0
187	29	1	61B1.1b,e	17	2	0	0	2	3	1
188	24	0	61C2.3c,d,m	26	0	0	0	3	4	1
189	49	0	61C2.3b,e,k	16	0	0	0	0	4	0
190	23	1	61B1.1	9	0	0	0	0	3	0
191	41	0	61B2.1b	22	0	2	3	2	3	1
192	27	1	61B2.1a,e	10	1	0	0	0	3	0
Excluded1	25	0	61B3.1b,d	66	5	1	4	0	5	0
Excluded2	50	1	61C1.3c	29	2	2	3	2	4	1
Excluded3	55	0	61B2.3d	27	3	0	3	2	3	1

Legend: AO OTA Classification is reported following Meinberg EG, Agel J, Roberts CS, Karam MD, Kellam JF, Fracture and Dislocation Classification Compendium-2018, J Orthop Trauma. 32 (2018) S1-S170. doi:10.1097/BOT.0000000000001063.

Highest AIS: each highest AIS score for every anatomical region is reported for every patient.

Excluded1 to Excluded3: indicates three patients excluded from the study. In two of them (Excluded2 & 3) the initial management was performed in another hospital. In the third patient (Excluded1), mechanical pelvic stabilization and angio-embolization were performed in the opposite sequence for an unknown reason.

The reuse of the repository data requires approval from both the senior author Dr. PD Axel Gamulin, and the Commission Cantonale d'Ethique de la Recherche, République et Canton de Genève (Ethics Committee).

Access to data of the Severely Injured Patients’ registry of the University Hospitals of Geneva not included in the repository may be discussed with the Senior Author upon reasonable request. Each new study project necessitates a new Ethics Committee approval.

**Table 2:** Pelvic ring injuries type A, B & C and concomitant injuries

AO OTA Classification	AIS head and neck ≥1	AIS face ≥1	AIS chest ≥1	AIS abdomen ≥1	AIS pelvis extremity ≥1	AIS external ≥1
A (38 patients)	25	7	31	23	38	6
B (125 patients)	66	22	79	62	125	18
C (32 patients)	15	7	22	27	32	6
Overall (195 patients)	106	36	132	112	195	30

Legend: AO OTA Classification is reported following Meinberg EG, Agel J, Roberts CS, Karam MD, Kellam JF, Fracture and Dislocation Classification Compendium-2018, J Orthop Trauma. 32 (2018) S1-S170. doi:10.1097/BOT.0000000000001063.

AIS ≥1 indicates an injury to the considered anatomical region. The number of patients with an AIS ≥1 is shown for each AIS region.

The reuse of the repository data requires approval from both the senior author Dr. PD Axel Gamulin, and the Commission Cantonale d'Ethique de la Recherche, République et Canton de Genève (Ethics Committee).

Access to data of the Severely Injured Patients’ registry of the University Hospitals of Geneva not included in the repository may be discussed with the Senior Author upon reasonable request. Each new study project necessitates a new Ethics Committee approval.

Table 1 shows age at the time of trauma, sex, and fracture classification according to the Arbeitsgemeinshaft für Osteosynthesefragen / Orthopaedic Trauma Association (AO OTA) [3], AIS scores and ISS of 195 high-energy blunt PRI patients from the SIPR.

The AO OTA classification distinguishes pelvic fractures into 3 main types - A, B, and C, depending on the degree of injury to the posterior arch. Type A represents fractures with intact posterior arch, B fractures with incomplete posterior arch disruption, and C fractures with complete posterior arch disruption. Each fracture type is further stratified into groups and subgroups.

Type A fractures are stable at the level of the posterior arch, such as avulsion of the innominate bone apophysis (group A1), fracture of the innominate bone (group A2) and transverse sacral fracture (group A3).

Type B and C fractures are considered unstable. Type B fractures involve the anterior arch and partial disruption of the posterior arch: unilateral with internal rotation displacement/rotational instability (group B1), unilateral with external rotation displacement/rotational instability (group B2), and bilateral with bilateral rotational instability (group B3). Type C fractures can have unilateral (group C1) and bilateral (group C3) complete disruption of the posterior arch provoking rotational and vertical instability, or bilateral involvement of the posterior arch which is incomplete on one hemipelvis and complete on the other one (group C2). For each type and group, there are multiple subgroups and qualifications according to the pelvic fracture pattern.

The AIS score describes the severity of injuries from 1 to 6 in the following body districts: head and neck, face, chest, abdomen, pelvis and extremities, and external (skin wounds, burns and other traumas). Zero indicates no injury for the specific body system.

The ISS score evaluates the most severe injuries from the aforementioned 6 body systems, distinguishing among no injury, minor, moderate, serious, severe, critical, and unsurvivable injury, obtaining overall scores from 0 to 75.

Out of these 195 patients, 192 were eligible for a study assessing the clinical outcomes of the institutional high-energy blunt PRI protocol [1]; three were not eligible because the patients had PRI management either at another institution (two cases) or not following the institutional protocol (one case).

Table 2 shows the number of patients presenting concomitant head and neck, face, chest, abdomen, pelvis and extremities, and external injuries for each fracture type A, B, C according to the AO OTA classification [3].

## 3 Experimental Design, Materials and Methods

The SIPR is an excel sheet including data on all high-energy trauma patients admitted to the University Hospitals of Geneva since mid-2013. This includes the following headings: demographics, prehospital and emergency department data (timing, vital parameters, accident details, imaging, fluid resuscitation, procedures), diagnosis, AIS and ISS, operations, intensive care management, outcome (mortality, post-acute care, complications). Around 300 items are recorded for each high-energy trauma patient,

A subset of SIPR patients present high-energy blunt PRI, defined as a closed fracture of the pelvic ring following falls from a height >1 m, road traffic accidents, sport, crush, farm and industrial injuries.

Data on this subset of patients are retrieved from the SIPR and collected in a separate high-energy blunt PRI patients database. Before the transfer of data from the SIPR to the high-energy blunt PRI patients database, patients’ identity is coded. The data linkage table is only available by the senior author, Dr. PD Axel Gamulin, and saved in the hospital server whose access is secured by nominal username and password. Data collected in the high-energy blunt PRI patients database include: sex, age, date of the accident, date and hour of admission to the Emergency Department, mechanism of injury, ISS and AIS codes, survival, death (death date), complications (type, number *per* patient, procedures required for management), intensive care unit hospitalization (yes/no and length of stay), overall in-hospital length of stay, hemodynamic instability at admission, number of packed red blood cells concentrates received within 24 hours from admission, pelvic fracture classification according to the AO OTA [3] and Young-Burgess classifications [4], emergency or urgent pelvic stabilization (type, date, timing), angioembolization (site of bleeding, site of embolization, timing), delayed surgery.

The dataset linked to this article reports the age, sex, pelvic fracture classification, AIS, and ISS of a cohort of 195 patients screened from the SIPR, studied by Valisena et al [1].

## Ethics Statements

The study was approved by the institutional research ethics board (Commission Cantonale d'Ethique de la Recherche, République et Canton de Genève, reference number 13-143R in 2013, re-approval reference number PB_2021-00045 in 2021). All procedures performed in studies involving human participants were in accordance with the ethical standards of the institutional research committee and with the 1964 declaration of Helsinki on medical research involving human subjects and its later amendments. For this type of study formal consent to participate was not required and was waived by the institutional research ethics board, even for minors under 18 years old.

## CRediT Author Statement

**Axel Gamulin, Silvia Valisena** and **Elisabeth Andereggen:** Conceptualization, Methodology, Investigation; **Alexandre Ansorge, Anna-Eliane Abboud** and **Silvia Valisena:** Data curation, Validation; **Axel Gamulin** and **Silvia Valisena:** Writing – original draft; **Axel Gamulin, Silvia Valisena, Elisabeth Andereggen, Alexandre Ansorge** and **Anna-Eliane Abboud:** Writing – review & editing.

## Declaration of Competing Interest

The authors declare that they have no known competing financial interests or personal relationships that could have appeared to influence the work reported in this paper.

## Data Availability

high-energy blunt pelvic ring injury (Original data) (Mendeley Data).
